# Large Bore Mechanical Thrombectomy for Right Atrial Thrombus and Pulmonary Embolism in the Early Post-Operative Period After Double Lung Transplantation

**DOI:** 10.1177/15269248241268689

**Published:** 2024-08-01

**Authors:** Blair E. Warren, Joyce Zaftis, Laura Donahoe, Jonathan Yeung, Abdul Aziz Qazi, Martin Urner, Sebastian Mafeld

**Affiliations:** 1 Joint Department of Medical Imaging, Division of Vascular and Interventional Radiology, 7989 University Health Network, Toronto, Ontario, Canada; 2 Division of Thoracic Surgery, Toronto General Hospital, 7989 University Health Network, 12366 University of Toronto, Toronto, Ontario, Canada; 3 Department of Anesthesiology and Pain Medicine, 12366 University of Toronto, Toronto, Canada; 4 Interdepartmental Division of Critical Care Medicine, 12366 University of Toronto, Toronto, Canada

**Keywords:** clinical outcomes, cardiovascular disease, quality, performance improvement, pathology, pulmonary ventilation, pharmacology, cardiovascular disease therapy, systems, health

## Introduction

Venous thromboembolism (VTE) is common following lung transplantation. A recent study examining the first year post transplant found 24.7% had VTE and 6% had pulmonary embolism.^1^ Pulmonary infarction following PE may be higher in lung transplant patients, due to absent bronchial artery collaterals.^2^ Intervention is recommended for pulmonary embolism with hemodynamic compromise and the presence of intra-cardiac thrombus further supports intervention.^3^ However, management of acute massive PE in the early post-operative setting post-lung transplant is a challenge, as thrombolytics or surgical thrombectomy come with significant challenges due to the bleeding risk from recent surgery. Large bore mechanical thrombectomy (LBMT) is an emerging technology that allows aspiration of thrombus without the use of thrombolytics.^3^ Little data exist on the use of LBMT in the setting of lung transplant, particularly across a recent vascular anastomosis. A case of LBMT for PE post lung transplant is reported, with consent from the patient.

## Presenting Concern

**Figure 1.**
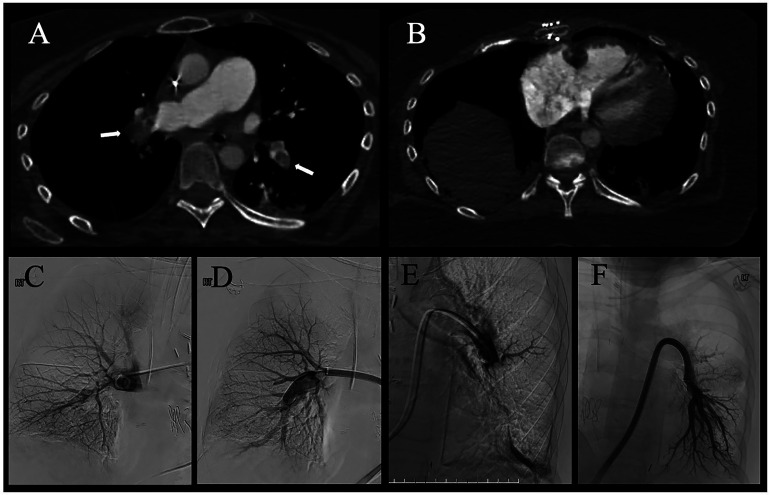
Computed tomography pulmonary angiogram and catheter pulmonary angiograms. (A) Extensive burden of pulmonary embolism bilaterally in the right and left pulmonary arteries extending into the lower lobes (arrows) with the right pulmonary vascular anastomosis seen, (B) enlarged right ventricle with increased RV:LV ratio and flattening of the interventricular septum. Right (C) and left (E) catheter pulmonary angiograms before LBMT demonstrating multiple filling defects, which are acute emboli and reduced pulmonary perfusion. Right (D) and left (F) catheter pulmonary angiograms after LBMT with resolution of the emboli and restoration of flow into the lower lobe vessels bilaterally.

A 41-year-old female underwent double lung transplantation 42 days prior and developed a massive PE requiring vasopressor support with hypotension, fluctuating levels of consciousness, and tachypnea. Upon transfer to the intensive care unit (ICU), a bedside echocardiogram was performed, showing a mobile right atrial mass: a suspected clot in transit. Computed tomography pulmonary angiogram confirmed bilateral lobar PE (**Figure 1A**) with right heart strain (**Figure 1B**).

## Clinical Findings

After multidisciplinary discussion with ICU and the transplant team, thrombolytics and surgical thrombectomy were considered high risk therefore endovascular LBMT was planned.

The LBMT was performed in a hybrid OR with fluoroscopy using the Inari Flowtriver system (Inari Medical Irvine, CA). The 24-French Inari Triever24 was advanced to the right atrium over a Glidewire (Terumo Medical, Tokyo, Japan) that was positioned in the right subclavian vein. Suction thrombectomy in the right atrium yielded acute thrombus. A 5Fr angled pigtail (Cordis Santa Clara, CA) was then navigated into the pulmonary artery (PA) and baseline PA pressures were obtained (66/23 mm Hg, mean of 38 mm Hg). The Inari Triever20 catheter was used bilaterally, and acute thrombus was aspirated (**Figure 1C-F**). Hemodynamic improvement was seen as thrombus burden was reduced. Completion angiography demonstrated marked improved perfusion bilaterally (**Figure 1D** and **F**) and PA pressures improved (38/15 mm Hg, mean of 23 mm Hg).

## Outcome

The post-procedure course was uncomplicated. An echocardiogram performed one day later demonstrated no residual right atrial thrombus and normal right ventricle function with a right ventricular systolic pressure of 31 mm Hg.

## Discussion

Pulmonary embolism is common post lung transplant however little data exist on the use of LBMT in these patients, with only one case report and a small case series of 8 patients.^4,5^ However, only 1 patient was <30 days post-surgery. The risks of passing a large bore 20- or 24-French catheter through a recently created vascular anastomosis are unknown. Nevertheless, given the risk of mortality and graft failure associated with the massive PE and right atrial thrombus, this case demonstrates the feasibility of LBMT in the clearance of cardiac and pulmonary thrombi in an early post-lung transplant patient.
